# Relationship between exercise capacity and fatigue, dyspnea, and lung function in non‐hospitalized patients with long COVID

**DOI:** 10.14814/phy2.15850

**Published:** 2023-11-20

**Authors:** Kiri Lovise Njøten, Birgitte Espehaug, Liv Heide Magnussen, Marte Jürgensen, Gerd Kvale, Eirik Søfteland, Bernt Bøgvald Aarli, Bente Frisk

**Affiliations:** ^1^ Department of Health and Functioning Western Norway University of Applied Sciences Bergen Norway; ^2^ Helse i Hardanger Øystese Norway; ^3^ Division of Psychiatry Haukeland University Hospital Bergen Norway; ^4^ Department of Clinical Psychology University of Bergen Bergen Norway; ^5^ Department of Medicine Haukeland University Hospital Bergen Norway; ^6^ Department of Clinical Science University of Bergen Bergen Norway; ^7^ Department of Thoracic Medicine Haukeland University Hospital Bergen Norway

**Keywords:** dyspnea, exercise capacity, fatigue, long COVID, lung function, post‐COVID‐19 condition

## Abstract

Long COVID is a global health problem that impairs patients' functional status. More than 200 reported symptoms have been identified where fatigue, dyspnea, and exercise impairment are most common. This study aimed to describe exercise capacity, fatigue, dyspnea, and lung function in previously non‐hospitalized patients with long COVID, and examine the relationship between exercise capacity and fatigue, dyspnea, and lung function. Sixty‐five patients, 54 women (83%), mean age of 39 standard deviation (12) years, were included and completed spirometry, cardiopulmonary exercise test, stair climbing test (SCT), 30 second sit‐to‐stand test (30STST), and questionnaires regarding fatigue and dyspnea. Fatigue was reported by 95% of the participants, whereas 65% reported severe fatigue, and 66% reported dyspnea. Mean exercise capacity measured with peak oxygen uptake (V̇O_2peak_ % pred.) was ≥85% in 65% of the participants. Mean forced expiratory volume in 1 s. and forced vital capacity were 96.6 (10.7)% and 100.8 (10.9)%, respectively, while reduced diffusion capacity for carbon monoxide (D_LCO_) was found in eight participants (13%). Reduced V̇O_2peak_ kg^−1^ and increased time on SCT were significantly associated with increased dyspnea and reduced D_LCO_ but not with fatigue, while 30STST was associated with increased fatigue and dyspnea in previously non‐hospitalized patients with long COVID.

## 1 INTRODUCTION

At least 65 million people worldwide are estimated to have long COVID (post‐COVID‐19 condition) (Davis et al., 2023), defined as long‐lasting symptoms 3 months after SARS‐CoV‐2 infection, persisting for at least 2 months with no other explanatory diagnosis (World Health Organization, 2022). The majority of the long COVID population consists of previously non‐hospitalized patients with a mild initial disease (Davis et al., 2023), and the incidence for long COVID of non‐hospitalized cases is estimated to 10%–30%, 50%–70% of the hospitalized (Bull‐Otterson et al., 2022; Ceban et al., 2022), and 10%–12% of the vaccinated (Al‐Aly et al., 2022; Ayoubkhani et al., 2022). More than 200 symptoms have been identified (Davis et al., 2023) with fatigue and dyspnea as the most frequently reported (Ma et al., 2022), but exercise impairments, palpitations, and mental health problems are also prevalent and impair patients' ability to work and maintain physical activity levels (Delbressine et al., 2021; Ladlow et al., 2023; Malik et al., 2022).

Our best indicator of cardiovascular fitness and exercise capacity is measuring the maximal amount of oxygen a person can utilize during maximal exercise by cardiopulmonary exercise testing (CPET), the peak oxygen uptake (V̇O_2peak_) (Radtke et al., 2019). Exercise capacity has previously been examined in long COVID patients, both in previously hospitalized and in non‐hospitalized patients, who have shown an overall normal peak oxygen uptake (Alba et al., 2021; Aparisi et al., 2022; Evers et al., 2022; Mancini et al., 2021; Schaeffer et al., 2022; Singh et al., 2022; Von Gruenewaldt et al., 2022). However, V̇O_2peak_ scores <85% predicted were found in approximately half of the participants with an initial mild disease in other studies (Jimeno‐Almazán et al., 2022; Rinaldo et al., 2021). In a Norwegian study, one‐third of previously hospitalized patients had reduced exercise capacity 3 months after the infection (Skjørten et al., 2021). The few studies that included only non‐hospitalized patients with long COVID have shown V̇O_2peak_ ranging between 83% and 117% of predicted values (Jimeno‐Almazán et al., 2022; Moulson et al., 2022; Rinaldo et al., 2021; Vonbank et al., 2021). In the study where a V̇O_2peak_ of 117% predicted was found, only young athletes with cardiopulmonary symptoms were included and tested 3 months after the initial infection. Deconditioning has been reported as the main reason for reduced exercise capacity (Moulson et al., 2022; Rinaldo et al., 2021; Skjørten et al., 2021; Vonbank et al., 2021), in addition to peripheral mechanisms, dysfunctional breathing, or hyperventilation (Singh et al., 2022; Skjørten et al., 2021; Von Gruenewaldt et al., 2022). The underlying pathophysiological mechanisms of long COVID conditions are unclear and can be complex and multifactorial. Organ damage from the acute infection may account for some of the symptoms. There can also be a long‐lasting inflammatory response and deconditioning following disease of a long duration very likely contributes to the condition. There is a wide range of sequelae affecting people with long COVID with varying physical condition. Even though the CPET is the gold standard for assessing exercise capacity (Palange et al., 2007), field tests such as walking tests, sit‐to‐stand tests, and stair climbing tests are also used to assess functional exercise capacity and include assessing of interactions between the respiratory, cardiovascular and musculoskeletal systems (Holland et al., 2014). Field tests are often more feasible than conducting a CPET to assess exercise capacity (Torres‐Castro et al., 2023).

In the acute phase of a COVID‐19 infection, the virus primarily invades pulmonary cells, often leading to respiratory symptoms, and can cause acute lung damage, especially in those who had a severe initial disease requiring hospitalization in an intensive care unit and ventilatory support (Wiersinga et al., 2020). However, the majority of patients with long COVID seem to have normal lung function according to predicted values for forced expiratory volume in 1 second (FEV_1_) and forced vital capacity (FVC), while diffusion capacity for carbon monoxide (D_LCO_) often is slightly reduced (Ma et al., 2022; Rinaldo et al., 2021; Vonbank et al., 2021). In addition, there are contradictory results regarding the initial severity of COVID‐19 and persistent impairment of D_LCO_ (Rinaldo et al., 2021, Vonbank et al., 2021).

Even though fatigue and dyspnea are comprehensive and debilitating symptoms in patients with long COVID (Ma et al., 2022), the relationship between exercise capacity, fatigue, dyspnea, and lung function is rarely investigated among previously non‐hospitalized patients with long COVID. In one of the few studies exploring this relationship, reduced exercise capacity was associated with increased dyspnea but not with fatigue (Jimeno‐Almazán et al., 2022). These complex long COVID symptoms include both physiological and psychological aspects (Hayen et al., 2013; Joli et al., 2022), and improved knowledge about the condition and the relationship between the most prevalent symptoms will broaden the understanding of the condition, which is important for providing tailored treatment to individuals.

The aims of the study were (1) to describe lung function, exercise capacity, fatigue, and dyspnea in previously non‐hospitalized patients with long COVID, and (2) examine the relationship between exercise capacity and possible influencing factors as fatigue, dyspnea, and lung function.

We hypothesized that reduced exercise capacity was associated with increased fatigue, dyspnea, and reduced lung function in previously non‐hospitalized patients with long COVID.

## 2 METHODS

### 2.1 Study design and participants

This cross‐sectional study was part of the prospective *Project Development of Smarter Health Solutions* study (the PUSH project) (Kvale et al., 2021), which is a collaboration between Haukeland University Hospital (Bergen, Norway) and the rehabilitation center, Helse i Hardanger (Øystese, Norway). Patients were referred by their general practitioners or other physicians to a concentrated 3‐day group rehabilitation. Non‐hospitalized patients between 18 and 67 years of age with long COVID and symptoms of sufficient severity that needed referral to specialist health care treatment were included in the study. Exclusion criteria were unstable chronic diseases where exercise training or physical activity was unsafe or not recommended, for example, major cardiovascular disorders. Patients with long COVID who while waiting to participate in the rehabilitation program, had improved functional status and reduced symptoms to an extent that they no longer needed rehabilitation, or who had received other treatment, were also excluded from participation. The patients had to be fluent in oral and written Norwegian and have sufficient digital competence to respond to online questionnaires. The current study reports baseline data.

### 2.2 Ethical considerations

The PUSH project was approved by the Norway Regional Committees for Medical and Health Research Ethics (REK 2020/101648), and the project is registered under Clinical Trials, identifier NCT05234281, with the approval date of 10/02/2022. Informed consent was obtained from all participants prior to inclusion. All measurements were performed in accordance with relevant guidelines and regulations.

### 2.3 Measurements

#### 2.3.1 Exercise capacity

*Cardiopulmonary exercise testing (CPET)* was conducted by having the patient walk uphill on a treadmill until exhaustion (Woodway, Würtzburg, Germany). The walking speed was individually set (ranging between 3.2 and 6.2 km/h), with an inclination of 0% at the start. The inclination was increased by 2% every 60 s up to 20%, followed by a 0.5 km h^−1^ increase in speed each minute until exhaustion. Continuous measurements of minute ventilation (V̇_E_), oxygen uptake (V̇O_2_), and carbon dioxide production (V̇CO_2_) were made by breath‐by‐breath sampling over 30 s intervals using a Hans Rudolph two‐way breathing mask (V2 mask, Shawnee, KS, USA). Heart rate (HR) was measured continuously using a 12‐lead electrocardiogram (ECG) (Custo Cardio 300, Custo Med, Ottobrun, Germany), and oxygen saturation (SpO_2_) was measured with an ear probe using a stationary pulse oximeter (Xpod, Nonin, MN, USA). All measurements were taken at rest, throughout the test, and for 2 min after termination. Blood pressure (Tango M2, SunTech Medical, Morrisville, NC, USA), perceived dyspnea, and leg fatigue were recorded using the Borg CR10 Scale (Borg, 1998) every second minutes throughout the test and at peak exercise. Reasons for termination other than exhaustion were pronounced pain or dizziness, ischemic ECG changes, or decreased systolic blood pressure below the resting pressure (Radtke et al., 2019). Reasons for ending the CPET were noted.

Normal values from a Norwegian reference population were used to interpret the CPET results (Edvardsen et al., 2013), and exercise capacity was reported as both V̇O_2peak_ kg^−1^ and V̇O_2peak_ % predicted to enable comparisons with other studies. A respiratory exchange ratio (RER) of >1.05 was considered a maximal test (Radtke et al., 2019). Desaturation was defined as a reduction in SpO_2_ ≥ 4% between rest and peak exercise, and SpO_2_ < 90% at peak exercise (Wedzicha, 1999). Maximal voluntary ventilation (MVV) was calculated using an estimate of FEV_1_ × 40, and breathing reserve was calculated as MVV − V̇_E_/MVV × 100 (Radtke et al., 2019). Ventilatory limitation was considered when breathing reserve was <15% (Weisman, 2003) and reduced ventilatory efficiency was defined by V̇_E_/V̇CO_2_ > 34 (Weisman, 2003). Circulatory limitation was considered if there were changes in the ECG consistent with ischemia or arrhythmia. V̇O_2peak_ < 85% was considered to indicate reduced exercise capacity with deconditioning or peripheral muscle limitations as explanatory causes if there were no ventilatory or circulatory exercise limitations (Radtke et al., 2019). Body mass index (BMI) was calculated by dividing weight by the square of height (kg m^−2^) with an accuracy of 0.5 cm and 0.1 kg (InBody 770, Seoul, South Korea).

*Stair climbing test (SCT)* was used to assess submaximal exercise capacity (Tveter et al., 2014). The participants were instructed to ascend and descend 18 steps three consecutive times as fast as possible. The patients could choose to walk or run but were not allowed to skip any steps. Time in seconds was the main outcome.

*Thirty‐second sit‐to‐stand test (30STST)* was used to assess lower extremity strength (Tveter et al., 2014). The participants started in a seated position on a chair, with their arms crossed, and were instructed to complete as many full stands as possible in 30 s. The number of repetitions was the main outcome.

#### 2.3.2 Fatigue

The *Chalder Fatigue Questionnaire (CFQ‐11)* (Chalder et al., 1993) was used to measure mental and physical fatigue. CFQ‐11 consists of 11 items, each scored between 0 and 3, with a maximum score of 33, where higher scores indicate more severe fatigue. It can also be calculated bimodally (0, 1) to provide a distinction between “cases” and “non‐cases” of fatigue where “better than usual” and “no worse than usual” are scored zero and “worse than usual” and “much worse than usual” are scored as one. A bimodal score of ≥4 was defined as a case of fatigue (Loge et al., 1998; Tack et al., 2020). Severe fatigue was defined as a bimodal score ≥ 4 and total score ≥ 23 (Blomberg et al., 2021).

#### 2.3.3 Dyspnea

The *Modified Medical Research Council (mMRC) scale* was used to measure dyspnea (Bestall et al., 1999). The scale is between zero and four, and higher scores indicate more severe symptoms. A cutoff ≥1, shortness of breath when hurrying on the level or walking up a slight hill, was used to define dyspnea (Jones et al., 2013).

#### 2.3.4 Lung function

*Spirometry* was conducted to measure lung function using a Vyntus Body/APS Plethysmograph (Vyaire Medical GmbH, Hochenberg, Germany). The highest FEV_1_ and FVC values from at least three satisfactory expiratory maneuvers were used. FVC and FEV_1_ values ≥80% and FEV_1_/FVC ≥70% predicted were considered normal (Johnson & Theurer, 2014). Procedures were performed according to the American Thoracic Society and European Respiratory Society guidelines (Graham et al., 2019). D_LCO_ was measured using an eight‐second single breath‐hold technique with a known tracer gas (Graham et al., 2017). The mean of two satisfactory test performances was used. A score >75% predicted was considered normal (Modi & Cascella, 2023).

### 2.4 Statistical analyses

Data were analyzed using IBM SPSS Statistics (version 28; IBM SPSS, Armonk, NY, United States of America). Descriptive statistics (mean, standard deviation [SD], median, and per cent) were used to describe the study population. Normality of data was assessed using histograms, Q–Q plots, and the Shapiro–Wilk test. Independent‐samples *t*‐tests were conducted to compare CPET variables and lung function between patients with V̇O_2peak_ < 85% of predicted values and those with V̇O_2peak_ ≥ 85%. Chi‐square tests were used to compare incidence of dyspnea and severe fatigue among patients with normal and reduced V̇O_2peak_.

The relationships between V̇O_2peak_ kg^−1^ and CFQ‐11 total score, mMRC total score, FEV_1_, D_LCO_, time since infection, age, sex, and BMI were examined in unadjusted and adjusted (multiple) linear regression analyses. Initially, we included all variables in the adjusted analyses (model 1), and secondly only variables with *p*‐values <0.2 (model 2). Analogous analyses were performed for time used in SCT and the number of repetitions in 30STST.

Estimated regression coefficients are presented with 95% confidence intervals (CI) and *p*‐values. The significance level was set at *α* = 0.05.

## 3 RESULTS

Of the 102 patients referred to the concentrated rehabilitation, 65 fulfilled the inclusion criteria. Reasons for exclusion were hospitalization owing to COVID‐19 (*n* = 18), improved long COVID symptoms (*n* = 16), and receiving other treatment (*n* = 3). Table 1 summarizes demographic and clinical characteristics. The mean age was 39 years, with a range of 19–65 years, and 83% of the participants were women. Seventy‐one per cent had higher education, and 83% had an active work status with 65% on full or partial sick leave at the time of inclusion. The mean duration of long COVID symptoms before inclusion was 9.4 months (range 3–24 months). Twelve (17%) participants had a BMI > 30 kg/m^2^ and six (8%) had a BMI > 35 kg/m^2^.

**TABLE 1:** Baseline characteristics of the study population at inclusion in the study.

Variables	Total *N* = 65
Female, *n* (%)	54 (83)
Age (years)	39.0 (11.8)
Height (m)	1.71 (0.08)
Weight (kg)	77.6 (16.0)
BMI (kg m^−2^)	26.5 (5.1)
Marital status, *n* (%)	
Single	19 (29)
Married/cohabiting	42 (65)
Girl‐/boyfriend/not cohabitating	4 (6)
Education, *n* (%)	
Lower secondary	0 (0)
Upper secondary	19 (29)
Higher	46 (71)
Working status, *n* (%)	
Employee	54 (83)
Student	10 (15)
Disability beneficiary	0 (0)
Pensioner	1 (2)
Sick leave at inclusion, *n* (%)	34 (65)
Time to baseline assessment since onset of disease (months)	9.4 (4.7)
Psychiatric illness, *n* (%)	
None	34 (52)
Previous psychiatric symptoms	17 (26)
Ongoing psychiatric symptoms	14 (22)

*Note*: Data are presented as mean ± SD unless otherwise stated.

Abbreviation: BMI, body mass index.

### 3.1 Exercise capacity, fatigue, dyspnea, and lung function

**FIGURE 1:**
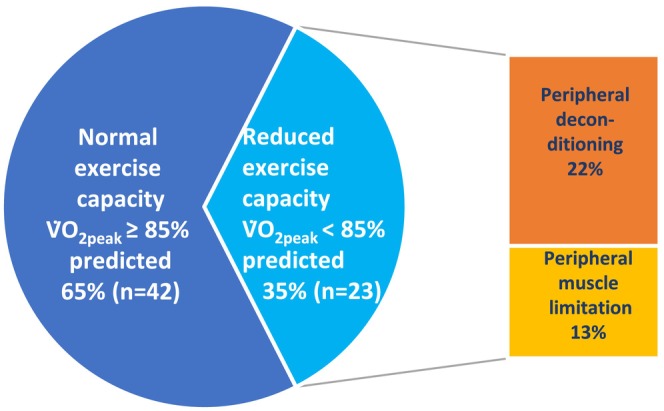
Exercise capacity and reasons for reduced peak oxygen uptake (V̇O_2peak_) (*n* = 65).

The mean V̇O_2peak_ % predicted was 92% (SD 16%) and V̇O_2peak_ kg^−1^ % predicted was 84% (SD 16%) (Table 2). V̇O_2peak_ < 85% predicted was present in 23 participants (35%) with deconditioning (65%) and peripheral muscle limitation (35%) as the main reasons for reduced exercise capacity (Figure 1). Fifty‐seven participants (86%) satisfied the maximal test criterion (RER > 1.05) and five (9%) had RER ≤ 1.05, indicating a submaximal test (Table 2). Three participants (5%) had a breathing reserve <15% indicating ventilatory limitation, and 14 participants (20%) had a V̇_E_/V̇CO_2_ slope > 34 indicating ventilatory inefficiency. None of the participants had circulatory limitations. Mean SpO_2_ at rest was 99% (SD 1%) and 96% (SD 3%) at peak exercise. Desaturation was registered in four participants (6%), who had a mean saturation at peak exercise at 87% (SD 1%). Oxygen pulse was significantly lower and breathing reserve higher in patients who had reduced exercise capacity compared to those with a normal exercise capacity (*p* < 0.001).

**TABLE 2:** Peak response to incremental exercise test on treadmill and lung function.

Variables	All *N* = 65 mean (SD)	V̇O_2peak_ % predicted ≥85% *N* = 42 mean (SD)	V̇O_2peak_ % predicted <85% *N* = 23 mean (SD)	*p*‐value
Time (min)	8.95 (1.60)	9.20 (1.56)	8.50 (1.61)	0.093
Performance				
V̇O_2peak_ (mL min^−1^)	2384 (483)	2547 (336)	2086 (569)	**<0.001**
V̇O_2peak_ (% pred)	91.7 (16.0)	100.6 (11.4)	75.3 (8.2)	**<0.001**
V̇O_2peak_ kg^−1^ (mL kg^−1^ min^−1^)	31.1 (6.4)	32.3 (5.2)	28.8 (7.7)	**0.029**
V̇O_2peak_ kg^−1^ % pred.	84.5 (15.7)	90.1 (13.4)	74.2 (14.6)	**<0.001**
V̇CO_2peak_ (mL min^−1^)	2747 (622)	2920 (417)	2432 (799)	**0.002**
Work rate (Watt)	252 (58)	272 (40)	214 (69)	**<0.001**
Dyspnea (Borg CR10)	8.5 (1.8)	8.8 (1.6)	7.9 (2.0)	0.069
Leg discomfort (Borg CR10)	7.7 (2.1)	7.7 (1.9)	7.8 (2.4)	0.793
Ventilation				
V̇_E peak_(L min^−1^)	86.8 (22.0)	92.2 (16.0)	76.7 (27.8)	**0.006**
MVV (L min^−1^)	133.0 (23.9)	131.6 (23.4)	135.7 (25.3)	0.516
Breathing reserve (% pred)	34.2 (14.1)	29.0 (10.4)	43.7 (15.3)	**<0.001**
Circulation				
HR_peak_ (beats min^−1^)	174.4 (15.5)	174.8 (16.1)	173.7 (14.5)	0.788
HR_peak_ (% pred)	95.2 (6.8)	96.2 (6.6)	93.2 (6.9)	0.089
Systolic BP (mmHg)	186 (32)	194 (31)	172 (28)	**0.007**
Diastolic BP (mmHg)	78 (15)	79 (16)	78 (15)	0.779
Oxygen pulse (mL stroke^−1^)	13.7 (2.8)	14.7 (2.3)	12.0 (2.8)	**<0.001**
Oxygen pulse (% pred)	97.9 (16.3)	106.0 (12.7)	81.5 (8.3)	**<0.001**
Gas exchange				
SpO_2_ rest (%)	99 (1)	99 (1)	98 (2)	0.203
SpO_2_ peak (%)	96 (3)	96 (4)	96 (3)	0.830
V̇_E_/V̇CO_2_ slope	31.6 (4.0)	31.5 (3.0)	31.7 (5.5)	0.870
RER	1.16 (0.09)	1.16 (0.07)	1.16 (0.13)	0.915
Lung function				
FEV_1_ (L)	3.3 (0.6)	3.3 (0.6)	3.4 (0.6)	0.516
FEV_1_ (% pred)	96.6 (10.7)	96.9 (10.7)	96.0 (11.0)	0.757
FVC (L)	4.2 (0.7)	4.3 (0.7)	4.2 (0.8)	0.762
FVC (% pred)	100.8 (10.9)	102.4 (10.8)	97.8 (10.6)	0.109
FEV_1_/FVC ratio (L)	78.8 (6.2)	77.5 (5.5)	81.1 (6.8)	**0.025**
FEV_1_/FVC (% pred)	95.7 (7.2)	94.5 (6.5)	98.0 (8.0)	0.056
TLC (L)	5.6 (1.0)	5.6 (0.9)	5.5 (1.1)	0.725
TLC (% pred)	97.7 (11.4)	99.3 (10.0)	95.3 (13.1)	0.201
Gas diffusion				
D_LCO_ (L)	8.4 (1.7)	8.5 (1.4)	8.2(2.1)	0.501
D_LCO_ (% pred)	87.4 (11.3)	90.3 (9.9)	82.1 (11.9)	**0.005**

*Note*: *p*‐values from independent‐samples *t*‐tests. Bold values: *p*<0.05.

Abbreviations: BP, blood pressure; D_LCO_, diffusion capacity of the lung for carbon monoxide; FEV_1_, forced expiratory volume in 1 second; FVC, forced vital capacity; HR, heart rate; MVV, maximal voluntary ventilation; RER, respiratory exchange ratio; SpO_2_, percutaneous oxygen saturation; TLC, total lung capacity; V̇CO_2_, carbon dioxide production; V̇_E_, minute ventilation; V̇O_2_, oxygen uptake.

The mean time used on the SCT was 43 s. Participants with normal V̇O_2peak_ were significantly faster in the SCT (40.8 s) than those with reduced exercise capacity (47.2 s) (*p* = 0.03). None of the participants experienced desaturation during the SCT. The mean number of repetitions in the 30STST was 19, and there were no statistically significant differences between those with V̇O_2peak_ < 85% and those with V̇O_2peak_ ≥ 85% (*p* = 0.932).

**FIGURE 2:**
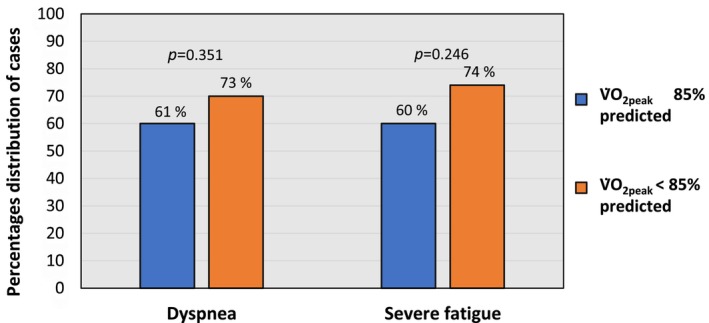
Patients with long COVID presented with dyspnea measured with Medical Research Council dyspnea scale (mMRC ≥ 1) and a peak oxygen uptake (V̇O_2peak_) ≥85% of predicted (*n* = 25, 61%) and reduced V̇O_2peak_ < 85% (*n* = 16, 73%), respectively, and severe fatigue measured with Chalder Fatigue Questionnaire (CFQ) bimodal score ≥ 4 and a total score ≥ 23, in those with normal (*n* = 25, 60%) and reduced V̇O_2peak_ (*n* = 17, 74%).

The mean CFQ‐11 total score was 24 (SD 5, range 12–33) indicating a high burden of fatigue. Fatigue, defined as a CFQ‐11 bimodal score ≥ 4, was present in 95% of the participants, while 65% fulfilled the criteria for severe fatigue (bimodal score ≥ 4 and total score ≥ 23). Dyspnea reported as mMRC ≥1 was present in 66% of the participants. There were no statistically significant differences between patients with normal and those with reduced V̇O_2peak_ regarding the burden of fatigue or dyspnea (*p* > 0.25) (Figure 2).

FEV_1_, FVC, and FEV_1_/FVC ratio were within predicted values (FEV_1_ 92%, FVC 97%, FEV_1_/FVC 100%) (Table 2). D_LCO_ ≤ 75% predicted was found in eight (13%) participants. D_LCO_ % predicted values were significantly lower in patients with V̇O_2peak_ < 85% predicted (*p* = 0.005).

### 3.2 Relationship of exercise capacity with fatigue, dyspnea, and lung function

A linear regression analysis of exercise capacity (Table 3, model 2) showed that V̇O_2peak_ kg^−1^ was negatively associated with mMRC (*B* = −1.81) and positively with D_LCO_ (*B* = 1.21). The same variables were associated with SCT, but with reversed directions. The 30STST score was negatively associated with CFQ‐11 total score (*B* = −0.39) and mMRC score (*B* = −3.66). In unadjusted regression analyses, FEV_1_ was associated with V̇O_2peak_ kg^−1^ and SCT, but the association was no longer present in the multivariate analyses.

**TABLE 3:** The relationship of exercise capacity with fatigue, dyspnea, and lung function in previously non‐hospitalized patients with long COVID.

Outcome	Unadjusted		Model 1				Model 2				
	*B*	*p*‐value	*B*	95% CI	*p*‐value	Adj. *R* ^2^	*B*	St. *B*	95% CI	*p*‐value	Adj. *R* ^2^
V̇O_2peak_ kg^−1^						0.49					0.52
CFQ‐11 total score	0.07	0.693	−0.08	−0.35–0.20	0.566						
mMRC total score	−1.73	0.072	−1.82	−3.75–0.10	0.063		−1.81	−0.24	−3.40−−0.22	**0.026**	
D_LCO_ (L)	1.52	**0.001**	1.23	−0.04–2.50	0.057		1.21	0.32	0.51–1.91	**0.001**	
FEV_1_ (L)	3.52	**0.007**	0.37	−3.25–3.98	0.840						
Time since infection	−0.09	0.621	−0.01	−0.28–0.25	0.913						
Age	−0.20	**0.003**	−0.16	−0.30−−0.03	**0.018**		−0.16	−0.30	−0.28−−0.05	**0.007**	
Sex	2.95	0.164	−0.73	−5.26–3.81	0.749						
BMI	−0.72	**<0.001**	−0.56	−0.84−−0.27	**<0.001**		−0.56	−0.44	−0.81−−0.30	**<0.001**	
SCT						0.34					0.35
CFQ‐11 total score	0.00	**0.997**	0.30	−0.27–0.88	0.294						
mMRC total score	3.88	**0.026**	3.21	−0.81–7.23	0.115		3.72	0.27	0.33–7.12	**0.032**	
D_LCO_ (L)	−2.57	**0.003**	−2.35	−5.00–0.30	0.081		−2.12	−0.31	−3.62−−0.62	**0.006**	
FEV_1_ (L)	−4.93	**0.038**	−2.39	−9.95–5.17	0.529						
Time since infection	−0.15	0.633	−0.33	−0.88–0.23	0.242						
Age	0.24	**0.045**	0.21	−0.07–0.49	0.131		0.23	0.23	−0.02–0.47	0.071	
Sex	−3.56	0.350	4.00	−5.48–13.48	0.401						
BMI	1.03	**<0.001**	0.79	0.19–1.38	**0.010**		0.73	0.32	0.19–1.28	**0.009**	
30STST						0.27					0.28
CFQ‐11 total score	−0.26	0.174	−0.32	−0.70–0.06	0.093		−0.39	−0.26	−0.73−−0.05	**0.025**	
mMRC total score	−3.25	**0.002**	−3.05	−5.70−−0.39	**0.025**		−3.66	−0.43	−5.70−−1.62	**<0.001**	
D_LCO_ (L)	1.08	0.051	0.73	−1.01–2.48	0.402						
FEV_1_ (L)	0.41	0.784	−3.09	−8.08–1.90	0.220						
Time since infection	0.19	0.311	0.17	−0.20–0.53	0.368						
Age	−0.05	0.544	−0.19	−0.37–0.00	**0.047**		−0.16	−0.26	−0.31−−0.01	**0.039**	
Sex	3.53	0.130	5.47	−0.78–11.72	0.085		4.75	0.26	0.70–8.79	**0.022**	
BMI	−0.40	**0.021**	−0.33	−0.72–0.06	0.096		−0.28	−0.21	−0.60–0.03	0.079	

Abbreviations: 30STST, 30 second sit‐to‐stand; 95% CI, 95% confidence interval; Adj. *R* ^2^, adjusted squared‐*R*; *B*, unstandardized regression coefficient; BMI, body mass index; CFQ‐11, Chalder Fatigue Questionnaire; D_LCO_, diffusion capacity % predicted; FEV_1_, forced expiratory volume in 1 second; FVC, forced vital capacity; mMRC, Modified Medical Research Council Dyspnea scale; SCT, stair climbing test; St. *B*, standardized regression coefficients; V̇O_2_, oxygen uptake. Note: Bold values: *p*<0.05.

## 4 DISCUSSION

The main findings in the current study of non‐hospitalized patients with long COVID were that even though near all participants suffered from fatigue, and a majority from dyspnea, the lung function was normal in 87%, and peak oxygen uptake normal in 65% of the participants. Reduced exercise capacity measured with CPET and stair climbing test were significantly associated with increased dyspnea and reduced gas diffusion, but not with fatigue. By contrast, leg strength measured by 30STST was significantly associated with fatigue and dyspnea, but not with lung function.

### 4.1 The characteristics of exercise capacity, fatigue, dyspnea, and lung function

Two‐thirds of the participants in our study had normal exercise capacity, which is in line with a Norwegian study of previously hospitalized COVID‐19 patients examined 3 months after discharge (Skjørten et al., 2021). Despite involving more severe cases of COVID‐19, older participants, and a higher proportion of men compared with our study, this may indicate that disease severity in the acute phase does not affect exercise capacity significantly. Vonbank et al. found a mean V̇O_2peak_ of 100% of predicted in patients with long COVID with an initial mild disease, suggesting a mainly normal exercise capacity (Vonbank et al., 2021). By contrast, V̇O_2peak_ < 85% predicted has been found in approximately half of the participants with an initial mild disease in other studies (Jimeno‐Almazán et al., 2022; Rinaldo et al., 2021). There was a lack of data regarding exercise capacity before infection in most published studies, including ours. Hence, even though still within the normal range, exercise capacity may have been reduced in some individuals compared with their level before infection as the range for the predicted values is wide, and a single measurement of exercise capacity will not cover a reduction in the individual's exercise capacity. It should also be noted that reduced exercise capacity in our study was calculated from V̇O_2peak_, which was higher than the predicted values of V̇O_2peak_ kg^−1^, reflecting the obesity among our participants. We also included two feasible field tests, SCT and 30STST. The validity and reliability had not been examined for patients with long COVID, but we choose to include them in our study to reveal additional information about the individuals' exercise capacity and these tests do not require any special equipment and are therefore easy to implement in clinical practice compared to CPET. We found that patients with V̇O_2peak_ kg^−1^ ≥ 85% were significantly faster on SCT than those with reduced V̇O_2peak_ kg^−1^, while there were no significant differences in number of repetitions on 30STST between the patients irrespective of V̇O_2peak_ kg^−1^. This is not surprising since the 30STST is measuring leg strength, while SCT is measuring both exercise capacity and leg strength and is therefore measuring more the same aspect of functional capacity as the CPET.

The CPET revealed that reasons for reduced exercise capacity in our study were deconditioning and peripheral muscle limitation. Although this has been found in some other studies (Rinaldo et al., 2021; Skjørten et al., 2021; Vonbank et al., 2021), the potential significance of this finding is unclear. Other studies report ventilatory inefficiency and abnormal ventilation during exercise as important factors for reduced exercise capacity in patients with a post‐COVID‐19 condition (Singh et al., 2022; Skjørten et al., 2021; Von Gruenewaldt et al., 2022). In contrast, we found ventilatory inefficiency in 20% and ventilatory limitation in 5% regardless of normal or reduced exercise capacity.

Interestingly, even though lung function was normal in 87% and exercise capacity in 65% of our participants, the majority still reported a high degree of fatigue and dyspnea which might indicate that these symptoms are not strongly related in patients suffering from long COVID. We found a high prevalence of fatigue and dyspnea which are in line with previous research showing that these symptoms are most commonly reported in patients with long COVID, even a year after the initial infection (Ma et al., 2022) and the prevalence >12 months after the initial disease have shown that 34% of participants reported fatigue and 29% dyspnea (Ma et al., 2022). In a Norwegian cohort including participants with initial mild COVID‐19 infection, regardless of persistent symptoms, the prevalence of fatigue was 30%, whereas severe fatigue (CFQ‐11 bimodal score ≥ 4 and total score ≥ 23) was present in 7% of previously non‐hospitalized patients and dyspnea reported by 15% 6 months after infection (Blomberg et al., 2021). As our study included participants based on persistent symptoms, a high prevalence of symptoms is expected and comparisons with the studies of Ma et al. and Blomberg et al. cannot be made (Blomberg et al., 2021; Ma et al., 2022). Jimeno‐Almazán et al. have similar inclusion criteria as in our study and found that the prevalence of fatigue (CFQ‐11 bimodal score ≥ 4) was 90% and dyspnea (mMRC ≥ 2) 43% for an average of 8 months after the initial disease. Even several months after initial infection, the magnitude and prevalence of fatigue and dyspnea found in our and previous studies may imply long‐lasting impairments in everyday life for a considerable number of people worldwide, regardless of the severity of the initial COVID‐19 disease.

### 4.2 The relationship between exercise capacity and fatigue, dyspnea, and lung function

We did find an association between V̇O_2peak_ kg^−1^ and reported dyspnea but not with fatigue. However, leg strength was associated with both fatigue and dyspnea. These findings are supported by those of Jimeno‐Almazán et al., where V̇O_2peak_ was associated with dyspnea and leg strength with fatigue (Jimeno‐Almazán et al., 2022). However, they used a submaximal CPET to assess V̇O_2peak_ and a five‐time sit‐to‐stand test and isometric knee extension test to measure leg strength (Jimeno‐Almazán et al., 2022). Hence, comparisons should be made with caution. We also found that reduced V̇O_2peak_ % pred. and increased time at SCT were significantly associated with increased dyspnea and reduced gas diffusion, but not with fatigue. To our knowledge, previous research has not examined these associations. Since the validity and reliability of SCT and 30STST have not been examined for patients with long COVID, conclusions regarding the usefulness cannot be made.

Our findings indicate that experiencing dyspnea may be more likely to affect exercise capacity than fatigue, even though both are complex phenomena (Hayen et al., 2013; Joli et al., 2022). It might be that the feeling of dyspnea is linked to dysfunctional breathing, as found in the study by von Gruenewaldt et al., which again impairs exercise capacity (Von Gruenewaldt et al., 2022). Dyspnea can also lead to early cessation of exercise (Hayen et al., 2013). Even though we found no significant difference in peak RER during CPET, there was a significantly lower oxygen pulse and increased breathing reserve in those not reaching 85% V̇O_2peak_, indicating that dyspnea might have led to a premature termination of the test.

Reduced D_LCO_ in patients with long COVID may indicate potential pulmonary fibrosis, interstitial lung damage, or incomplete recovery (Cherrez‐Ojeda et al., 2022; Munker et al., 2022), impairing the lungs' ability to “transfer” oxygen to blood (Modi & Cascella, 2023). While spirometry measurements often remain well preserved, the most common abnormality found in people recovering from COVID‐19 infection is a reduced D_LCO_, with prevalence increasing with the severity of the initial disease (Thomas et al., 2021). Hence, impaired D_LCO_ could be a reason for reduced exercise capacity. In our study, we found that reduced exercise capacity was significantly associated with impaired D_LCO_, even though mean D_LCO_ was 87% of predicted and only eight participants had D_LCO_ < 75%. Our finding is supported by published studies of both previously hospitalized and non‐hospitalized patients where mean D_LCO_ was 85%–87% of predicted values and was related to V̇O_2peak_ (Johnsen et al., 2021; Vonbank et al., 2021). By contrast, it was also suggested that there is no correlation between D_LCO,_ and exercise capacity measured with a 6‐minute walk test (Cherrez‐Ojeda et al., 2022). In addition, the impairment of D_LCO_ seems to be reversible (Ma et al., 2022), and other factors are likely to play a role in reduced exercise capacity in patients with long COVID.

Time since initial COVID‐19 infection and assessment in our study varied between 3 and 24 months. Even with an almost two‐year difference, we did not find any association between time and exercise capacity. To our knowledge, no other studies have examined time since infection and V̇O_2peak_. A study using a submaximal CPET found that those who more recently had a COVID‐19 infection were more likely to not achieve 85% of maximum heart rate (HR_max_) (Romero‐Ortuno et al., 2022). Comparisons should be made with caution because the main outcome was the ability to reach 85% of HR_max_, calculated based on age and not V̇O_2peak_ as in our study and others. The lack of association between the time since infection and V̇O_2peak_ could be explained by the relatively normal exercise capacity found in our study. However, it could also indicate that exercise capacity does not automatically improve, even though symptoms such as fatigue and dyspnea are shown to improve over time (Ma et al., 2022).

### 4.3 Strengths and limitations

A strength of this study was the thorough examination of patients. Furthermore, the inclusion of only non‐hospitalized patients with long COVID made the study population more homogenous. Most published studies include both previously hospitalized and non‐hospitalized patients with long COVID. Owing to differences in the acute severity, a different course of illness could be expected. Only including those with initially mild disease, confines the patient group which ensures clearer results. In addition, using these inclusion criteria allows comparisons with previous studies from a similar geographical area (Blomberg et al., 2021; Fjelltveit et al., 2023).

Because the current study has a cross‐sectional design, and exposure and outcome are measured simultaneously, causality cannot be determined. The sample size in this study is relatively small; however, previously published comparable studies have similar sample sizes.

## 5 CONCLUSION

Our findings showed that non‐hospitalized patients with long COVID evaluated 9 months after the initial disease had an overall normal exercise capacity and lung function despite reporting a high impact of fatigue and dyspnea. Deconditioning and peripheral muscle limitations were the main reasons for reduced exercise capacity. Reduced peak oxygen uptake was associated with increased dyspnea and reduced gas diffusion but not with fatigue.

## AUTHOR CONTRIBUTIONS

Bente Frisk, Marte Jürgensen, Gerd Kvale, Kiri Lovise Njøten, Eirik Søfteland, Birgitte Espehaug, Bernt Bøgvald Aarli, and Liv Heide Magnussen made substantial contributions to the conception and design of the study. Kiri Lovise Njøten was in charge of data collection. Kiri Lovise Njøten, Birgitte Espehaug, Liv Heide Magnussen, and Bente Frisk performed data analysis and contributed to data interpretation. Kiri Lovise Njøten and Bente Frisk drafted the initial manuscript, which was subsequently revised by all authors and all authors approved the final manuscript.

## FUNDING INFORMATION

This study was part of the PUSH project which was funded by Helse in Hardanger and Bergen Hospital Trust.

## CONFLICT OF INTEREST STATEMENT

Kiri Lovise Njøten, Gerd Kvale, Marte Jürgensen, Liv Heide Magnussen, Birgitte Espehaug, Bente Frisk, and Eirik Søfteland have nothing to disclose. Bernt Bøgvald Aarli has received grants, consulting fees or honoraria from Boehringer Ingelheim, GlaxoSmithKline, Astra Zeneca, Novartis, and Sanofi‐Aventis.

## Supporting information


Data S1.

## Data Availability

In accordance with the approvals granted for this study by the Regional Committee on Medical Research Ethics and the Norwegian Data Inspectorate, the data files will be stored securely and in accordance with the Norwegian Law of Privacy Protection. A subset of the data file with anonymized data will be made available to interested researchers upon reasonable request to Bente Frisk: bente.frisk@hvl.no, providing that Norwegian privacy legislation and the General Data Protection Regulation are respected, and that permission is granted from the Norwegian Data Inspectorate and the data protection officer at Haukeland University Hospital.
