# Supplementary material for: Relationship between exercise capacity and fatigue, dyspnea, and lung function in non‐hospitalized patients with long COVID
Source: Physiol Rep. 2023 Nov 20;11(22):e15850. doi: 10.14814/phy2.15850 (PMC10659919; doi:10.14814/phy2.15850)
Supplement: Supplementary file 1 — Data S1. [file PHY2-11-e15850-s001.docx]

The second wave of COVID consequences is hitting hard at the moment, i.e. the long COVID / post COVID-19 condition. The societal and personal costs are huge, with an estimated 2-4 million people unable to work in the US alone [1]. The CDC estimates that 1 in 5 adults who had COVID-19 infection suffer from Long COVID symptoms, whereas a Norwegian study recently published in Nature Medicine indicated that even in young adults with relatively mild primary infection, 52% had symptoms at 6 months [2, 3].

Healthcare providers are in desperate need to find a treatment that improves the quality of life and ability to work in patients with long COVID. However, this is a challenging task as long COVID is a complex phenomenon with over 200 reported symptoms [4]. The lungs have been reported to be affected [5] while reduced exercise capacity have been shown in one third of previously hospitalized patients with long COVID [6]. The most reported long COVID symptoms are fatigue and dyspnea [7].

We hereby present the results from a cross sectional study on previously non-hospitalized patients with long COVID in mean nine months after the initial infection where the relationship between exercise capacity and fatigue, dyspnea and lung function have been examined. All patients have conducted cardiopulmonary exercise test, spirometry including diffusion capacity for carbon monoxide and answered validated questionnaires regarding fatigue and dyspnea. The findings may help increase our knowledge regarding pulmonary and cardiorespiratory manifestations as well as potential relationships with frequently reported symptoms of long COVID which may further be of importance in developing treatment for the patient group.

**REFERENCES**

1. Brookings Institue. Long COVID keeping 4 mill people out of work. 2022. Available from: <https://www.brookings.edu/research/new-data-shows-long-covid-is-keeping-as-many-as-4-million-people-out-of-work/>.

2. CDC. Nearly One in Five American Adults Who Have Had COVID-19 Still Have “Long COVID.” 2022.

3. Blomberg B, Mohn KG, Brokstad KA, Zhou F, Linchausen DW, Hansen BA, Lartey S, Onyango TB, et al. Long COVID in a prospective cohort of home-isolated patients. Nat Med 2021;27(9):1607-1613.

4. Davis HE, McCorkell L, Vogel JM, Topol EJ. Long COVID: major findings, mechanisms and recommendations. Nature Reviews Microbiology 2023:1-14.

5. Wiersinga WJ, Rhodes A, Cheng AC, Peacock SJ, Prescott HC. Pathophysiology, transmission, diagnosis, and treatment of coronavirus disease 2019 (COVID-19): a review. Jama 2020;324(8):782-793.

6. Skjørten I, Ankerstjerne OAW, Trebinjac D, Brønstad E, Rasch-Halvorsen Ø, Einvik G, Lerum TV, Stavem K, et al. Cardiopulmonary exercise capacity and limitations 3 months after COVID-19 hospitalisation. European Respiratory Journal 2021;58(2).

7. Ma Y, Deng J, Liu Q, Du M, Liu M, Liu J. Long-Term Consequences of COVID-19 at 6 Months and Above: A Systematic Review and Meta-Analysis. International Journal of Environmental Research and Public Health 2022;19(11).
